# Observations on the State of Health of the Troops in North Britain, during a Period of Seven Years, from 1816 to 1822, Both Inclusive

**Published:** 1823-10

**Authors:** Henry Marshall

**Affiliations:** Surgeon to the Forces, &c.


ART. IV.—*Observations on the State of Health of the Troops in North Britain, during a period of Seven Years, from* 1816 *to* 1822, *both inclusive.* By HENRY MARSHALL, Esq. Surgeon to the Forces, &c.

IT has ever been an object of zealous research with medical inquirers to ascertain the exact extent of the existence of individual diseases in particular situations, the ratio of fatality by each disease, and the proportional mortality which occurs among the population of a certain district of country, or class of people. Statements of this kind possess much interest, by affording the means of comparing the prevalence of diseases, and their consequences, in different parts of the world, or even in the same district at different periods. In civil life, the requisite data for compiling returns sufficiently exact and comprehensive to fulfil the above desirable objects, can rarely or never be procured. Much better opportunities are afforded for compiling correct statistical statements among certain classes of people, more particularly of the army. Military returns possess a precision superior, perhaps, to all others, on account of the exactness with which the strength may be ascertained; as also the results of disease, whether these happen to be recovery or death.

Presuming that an interesting statement might be compiled from the returns of the sick of the troops employed in North Britain, during a period of seven years,—or from the 21st December, 1815, to the 20th December, 1822,—I have, in the following tables, exhibited a numerical view of the health of the army in Scotland during that period.

These tables possess some interest, by showing the diseases which occur among individuals in this country during the prime of life, and the comparative frequency of particular complaints during distinct periods of time.

The advantages which the science of medicine may derive from pathological views drawn from great masses of people, by tables of this kind, are so obvious as not to require to be more particularly stated. Sir Gilbert Blane, in his interesting paper "on the Prevalence of different Diseases in London," has well observed, that, "as all practical researches ought to be built upon an induction of facts, single objects or events are of little value, except in so far as they stand related to others."

Table No. I. is arranged according to the order of the "Table of Diseases for Medical Returns," and exhibits in seven columns the number of patients of each disease treated in the Military Hospitals in Scotland for seven years, and the absolute and relative mortality by each disease.

TABLE, No. I.

ABSTRACT *of the* RETURNS *of the* SICK *of the* TROOPS *employed in* NORTH BRITAIN, *from* 1816 *to* 1822 *inclusive.*

| DISEASES. | *Number of Patients treated in the Hospitals.* | | | | | | | *Total treated.* | *Died.* |
|---|---|---|---|---|---|---|---|---|---|
| | 1816 | 1817 | 1818 | 1819 | 1820 | 1821 | 1822 | | |
| Febris Intermittens | 179 | 20 | 2 | 9 | 27 | 2 | 5 | 244 | 2 |
| ——— Remittens | .. | .. | .. | .. | .. | 1 | .. | 1 | |
| ——— Continua Communis | 136 | 120 | 193 | 151 | 399 | 121 | 145 | 1265 | 34 |
| ——— Synochus | .. | 1 | .. | .. | .. | .. | .. | 1 | |
| ——— Typhus | 20 | 3 | 1 | 4 | 4 | 1 | 2 | 35 | 11 |
| Phlegmon | .. | 107 | 100 | 73 | 184 | 87 | 78 | 629 | 1 |
| Purunculus | .. | .. | .. | .. | .. | .. | 6 | 6 | |
| Paronychia | .. | .. | 10 | 3 | 6 | 1 | 2 | 22 | |
| Morbi Ocularum | 107 | 85 | 92 | 41 | 166 | 137 | 68 | 696 | 1 |
| Phrenitis | .. | .. | .. | 1 | .. | 1 | .. | 2 | 1 |
| Cephalalgia | .. | .. | 3 | 1 | .. | 5 | 1 | 10 | 1 |
| Inflammatio Cerebri | .. | .. | .. | .. | .. | 1 | .. | 1 | 1 |
| Contusio Cerebri | .. | .. | .. | 1 | .. | .. | .. | 1 | 1 |
| Cynanche Tonsillaris | .. | 26 | 33 | 38 | 51 | 37 | 17 | 202 | |
| ——— Maligna | .. | .. | .. | .. | .. | 1 | .. | 1 | |
| ——— Parotidea | .. | .. | 3 | 7 | 18 | 1 | 1 | 30 | |
| ——— Trachealis | .. | .. | .. | .. | 1 | .. | .. | 1 | |
| ——— Laryngea | .. | .. | .. | .. | 2 | .. | .. | 2 | 2 |
| Pneumonia | 107 | 62 | 74 | 67 | 167 | 81 | 54 | 612 | 22 |
| Pleuritis | 1 | 1 | .. | .. | 1 | 3 | .. | 6 | 2 |
| Carditis | .. | .. | .. | .. | 1 | .. | .. | 1 | |
| Pericarditis | .. | 1 | .. | .. | .. | .. | .. | 1 | 1 |
| Gastritis | .. | 1 | .. | .. | 1 | 1 | .. | 3 | 2 |
| Enteritis | .. | 1 | .. | 1 | 3 | 2 | 1 | 8 | 5 |
| Hepatitis Acuta | 12 | 7 | 7 | 2 | 13 | 5 | 1 | 47 } | 5 |
| ——— Chronica | 10 | 7 | 1 | 3 | 7 | 11 | 1 | 40 } | |
| Splenitis | .. | .. | 2 | .. | 3 | 8 | 3 | 16 | |
| Nephritis | .. | .. | 1 | 3 | 1 | 1 | .. | 6 | 1 |
| Cystitis | .. | .. | .. | 2 | 2 | 1 | .. | 5 | 1 |
| Otitis | .. | 2 | 3 | .. | 6 | 5 | .. | 16 | |

| DISEASES. | *Number of Patients treated in the Hospitals.* | | | | | | | *Total treated.* | *Died.* |
|---|---|---|---|---|---|---|---|---|---|
| | 1816 | 1817 | 1818 | 1819 | 1820 | 1821 | 1822 | | |
| Rheumatismus Acutus | 42 | 29 | 49 | 47 | 95 | 69 | 17 | 348 | 3 |
| ——— Chronicus | 41 | 25 | 49 | 38 | 87 | 72 | 46 | 358 | |
| Arthritis | .. | .. | 1 | .. | .. | 1 | .. | 2 | |
| Variola | 3 | 3 | 10 | .. | 13 | 3 | .. | 32 | 3 |
| Vaccina | .. | 3 | .. | .. | .. | .. | .. | 3 | |
| Varicella | .. | .. | 1 | 3 | .. | 1 | .. | 5 | |
| Rubeola | 3 | 1 | 7 | 10 | .. | 10 | 1 | 32 | 1 |
| Scarlatina | .. | .. | 1 | .. | 2 | 1 | .. | 4 | |
| Erysipelas | 4 | .. | 4 | 8 | 4 | 11 | 6 | 37 | 4 |
| Urticaria | .. | .. | 3 | 2 | 2 | .. | .. | 7 | |
| Epistaxis | .. | .. | 1 | .. | 1 | .. | .. | 2 | |
| Hæmoptysis | .. | 6 | 4 | 9 | 7 | 6 | 4 | 36 | 3 |
| Hæmatemesis | .. | .. | .. | .. | .. | 1 | .. | 1 | |
| Phthisis Pulmonalis | 25 | 24 | 18 | 13 | 25 | 24 | 21 | 150 | 56 |
| Hæmorrhois | .. | 11 | 11 | 8 | 45 | 11 | 7 | 93 | |
| Catarrhus Acutus | 139 | 50 | 55 | 128 | 254 | 160 | 83 | 869 | 13 |
| ——— Chronicus | 188 | 47 | 7 | 2 | 57 | 42 | 2 | 345 | |
| Dysenteria | 4 | 14 | 4 | 1 | 14 | 5 | .. | 42 | |
| Apoplexia | 3 | 3 | 2 | 5 | 1 | 1 | 2 | 17 | 8 |
| Paralysis | .. | 1 | .. | 1 | 3 | 11 | 4 | 20 | |
| Dyspepsia | .. | 1 | 7 | 3 | 7 | 3 | 4 | 25 | |
| Tetanus | 1 | .. | .. | .. | .. | .. | .. | 1 | 1 |
| Epilepsia | 4 | 1 | 3 | .. | 7 | 2 | .. | 17 | 3 |
| Asthma | .. | 1 | 4 | 8 | 27 | 22 | 1 | 63 | 2 |
| Dyspnœa | .. | .. | 6 | 2 | 5 | 1 | .. | 14 | |
| Colica | .. | 1 | 10 | 7 | 19 | 2 | 8 | 47 | |
| Cholera Morbus | .. | 6 | 11 | 5 | 24 | 8 | 10 | 64 | 1 |
| Diarrhœa | 65 | 34 | 39 | 39 | 80 | 57 | 29 | 343 | 3 |
| Diabetes | .. | 1 | 2 | .. | .. | .. | .. | 3 | 1 |
| Amentia | .. | .. | .. | .. | .. | 1 | .. | 1 | |
| Mania | .. | .. | 1 | .. | 4 | 3 | 1 | 9 | |
| Delirium Tremens | .. | .. | 2 | .. | .. | 1 | .. | 3 | |
| Atrophia | .. | 1 | 1 | .. | .. | 1 | 1 | 4 | 1 |
| Anasarca | .. | 5 | 2 | .. | 2 | 3 | 1 | 13 | 2 |
| Tympanitis | .. | .. | .. | .. | .. | .. | 1 | 1 | |
| Hydrocephalus | .. | .. | .. | 1 | .. | .. | .. | 1 | 1 |
| Hydrothorax | 1 | 1 | 1 | 1 | 4 | 1 | .. | 9 | 8 |
| Ascites | 1 | 1 | 3 | 5 | 3 | 2 | 2 | 17 | 6 |
| Hydrops | 13 | 5 | .. | .. | .. | .. | .. | 18 | 1 |
| Hydrocele | .. | .. | 1 | 3 | 3 | 4 | 5 | 16 | |
| Vermes | .. | 4 | 3 | 1 | 8 | 2 | .. | 18 | |
| Scrophula | .. | .. | 5 | 1 | 11 | 14 | 2 | 33 | |
| Hydarthrus | .. | .. | 3 | .. | 1 | .. | 1 | 5 | |
| Morbus Coxarius | .. | .. | 2 | 1 | .. | .. | .. | 3 | 1 |
| Apostema Lumbare | 1 | .. | .. | .. | .. | .. | 1 | 2 | 1 |
| Syphilis Primitiva | .. | .. | 253 | 146 | 196 | 145 | 76 | 816 | |
| ——— Consecutiva | .. | .. | 18 | 25 | 44 | 18 | 113 | 218 | |
| Morbi Venerei | 433 | 494 | 43 | .. | .. | .. | .. | 970 | 1 |
| Ulcera Penis non Syphilit. | .. | .. | 40 | 125 | 264 | 129 | 41 | 599 | |
| Bubo Simplex | .. | .. | 29 | 38 | 52 | 19 | 19 | 157 | |
| Cachexia Syphiloidea | .. | .. | 7 | .. | 4 | .. | .. | 11 | |
| Phymosis | .. | .. | 1 | 1 | 1 | 1 | .. | 4 | |
| Paraphymosis | .. | .. | .. | .. | 2 | .. | .. | 2 | |
| Erythema Mercurialis | .. | .. | 2 | .. | .. | .. | .. | 2 | |
| Scorbutus | .. | .. | 2 | 1 | .. | .. | .. | 3 | |
| Icterus | .. | .. | 6 | 10 | 12 | 7 | 3 | 38 | |

| DISEASES. | Number of Patients treated in the Hospitals. | | | | | | | Total treated. | Died. |
|---|---|---|---|---|---|---|---|---|---|
| | 1816 | 1817 | 1818 | 1819 | 1820 | 1821 | 1822 | | |
| Dysecæa | .. | .. | 2 | 1 | 3 | .. | .. | 6 | |
| Contractura | .. | 2 | 3 | .. | .. | .. | .. | 5 | |
| Eneuresis | .. | 1 | 1 | 1 | 3 | 1 | .. | 7 | |
| Gonorrhœa | .. | .. | 114 | 112 | 235 | 79 | 82 | 622 | |
| Hernia Humoralis | .. | .. | 24 | 50 | 60 | 37 | 29 | 200 | |
| Strictura Urethræ | .. | .. | .. | 2 | 9 | 4 | 2 | 17 | |
| Sarcocele | .. | .. | 2 | 1 | 1 | .. | .. | 4 | |
| Obstipatio | .. | .. | 2 | .. | 2 | 2 | 2 | 8 | |
| Ischuria | 1 | .. | 2 | 1 | 2 | 1 | .. | 7 | |
| Dysuria | .. | 1 | 2 | 2 | 1 | 2 | 1 | 9 | |
| Calculus Vesicæ | .. | 1 | .. | .. | .. | .. | .. | 1 | |
| Varix | .. | .. | 4 | .. | .. | 1 | .. | 5 | |
| Schirrhus | .. | .. | 2 | 1 | .. | .. | .. | 3 | |
| Tumores | .. | .. | 6 | 1 | 15 | 13 | .. | 35 | 1 |
| Verrucæ | .. | .. | 2 | 4 | 3 | 10 | 2 | 21 | |
| Hernia | 1 | 2 | 3 | 1 | 1 | .. | .. | 8 | |
| ——— Inguinalis | .. | .. | .. | .. | .. | 1 | 1 | 2 | |
| Prolapsus Ani | .. | .. | .. | .. | 1 | 1 | .. | 2 | |
| Fistula in Ano | .. | .. | 1 | .. | 2 | 1 | 2 | 6 | |
| ——— in Perinæo | .. | .. | .. | .. | 1 | .. | .. | 1 | |
| ——— Lachrymalis | .. | .. | 1 | .. | .. | .. | .. | 1 | |
| Luxatio | .. | 1 | 2 | 3 | 5 | 1 | 1 | 13 | |
| Subluxatio | .. | 8 | 21 | 6 | 20 | 12 | 1 | 68 | |
| Vulnus | 52 | 32 | 48 | 45 | 55 | 24 | 28 | 284 | 1 |
| Contusio | 245 | 134 | 132 | 83 | 149 | 136 | 107 | 986 | 2 |
| Ambustio | .. | .. | 12 | 2 | 6 | 6 | 1 | 27 | |
| Irelatio | .. | .. | 2 | .. | .. | .. | .. | 2 | |
| Ulcus | 345 | 149 | 144 | 106 | 286 | 168 | 81 | 1279 | 3 |
| Necrosis | 1 | .. | .. | 1 | 2 | 2 | 2 | 8 | |
| Fractura | 1 | 4 | 7 | 11 | 14 | 6 | 1 | 44 | 4 |
| Amputatio | .. | 1 | .. | .. | .. | 1 | 1 | 3 | |
| Punitus | 29 | 68 | 69 | 83 | 138 | 67 | 29 | 483 | |
| Morbi Cutis | 588 | 404 | 413 | 158 | 506 | 124 | 87 | 2280 | |
| Morbi Chronici | 63 | 7 | .. | .. | .. | .. | .. | 70 | |
| Varii | .. | .. | 1 | .. | 7 | 3 | .. | 11 | |

For the purpose of exhibiting the comparative frequency with which particular organs and parts of the body become affected by disease, I have, in the subjoined table, arranged the diseases under nine classes.

TABLE, No. II.

| Class. | Diseases. | No. treated of each Disease. | Died. | Proport. of Deaths to No. treated. | No. of Deaths in each Class |
|---|---|---|---|---|---|
| 1. Constitutional Affections.. .. | Febris Intermittens | 244 | 2 | 1 in 122 | 64 |
| | ——— Remittens .. | 1 | | | |
| | ——— Contin. com. | 1265 | 34 | 1 in 37 | |
| | ——— Synochus .. | 1 | | | |
| | ——— Typhus .... | 35 | 11 | 1 in 11$\frac{2}{11}$ | |
| | Rheumatismus Acut. | 348 | 3 | 1 in 235 | |
| | ——— Chronicus | 358 | | | |
| | Arthritis .......... | 2 | | | |
| | Variola .......... | 32 | 3 | 1 in 10$\frac{2}{10}$ | |
| | Varicella.......... | 5 | | | |
| | Vaccina .......... | 3 | | | |
| | Rubeola .......... | 32 | 1 | 1 in 32 | |
| | Scarlatina ........ | 4 | | | |
| | Erysipelas ........ | 37 | 4 | 1 in 9$\frac{1}{4}$ | |
| | Urticaria.......... | 7 | | | |
| | Syphilis Consecutiva | 218 | | | |
| | Morbi Venerii .... | 970 | 1 | 1 in 970 | |
| | Cachexia Syphiloida | 11 | | | |
| | Erythema Mercur. | 2 | | | |
| | Scorbutus ........ | 3 | | | |
| | Morbi Chronici .... | 70 | | | |
| | Varii ............ | 11 | | | |
| | Tetanus .......... | 1 | 1 | 1 in 1 | |
| | Atrophia.......... | 4 | 1 | 1 in 4 | |
| | Anasarca.......... | 13 | 2 | 1 in 6$\frac{1}{2}$ | |
| | Tympanitis........ | 1 | | | |
| | Hydrops .......... | 18 | 1 | 1 in 18 | |
| | Scrophula ........ | 33 | | | |
| 2. D. of the Skin | | 2280 | | | |
| 3. Diseases of the Head .... | Phrenitis .......... | 2 | 1 | 1 in 1 | 16 |
| | Inflammatio Cerebri | 1 | 1 | 1 in 1 | |
| | Contusio Cerebri .. | 1 | 1 | 1 in 1 | |
| | Apoplexia ........ | 17 | 8 | 1 in 2$\frac{1}{8}$ | |
| | Paralysis.......... | 20 | | | |
| | Epilepsia.......... | 17 | 3 | 1 in 5$\frac{2}{3}$ | |
| | Amentia .......... | 1 | | | |
| | Mania ............ | 9 | | | |
| | Delirium Tremens.. | 3 | | | |
| | Hydrocephalus .... | 1 | 1 | 1 in 1 | |
| | Dysecæa.......... | 6 | | | |
| | Cephalalgia ...... | 9 | 1 | 1 in 9 | |
| 4. D. of the Eyes | | 696 | 1 | 1 in 696 | 1 |
| 5. Diseases of the Throat .. | Cynanche Tonsillaris | 202 | | | 2 |
| | ——— Maligna .. | 1 | | | |
| | ——— Parotidea | 30 | | | |
| | ——— Trachealis | 1 | | | |
| | ——— Laryngea | 2 | 2 | 1 in 1 | |
| 6. Diseases of the Thorax .. | Pneumonia........ | 612 | 22 | 1 in 27 | 107 |
| | Pleuritis .......... | 6 | 2 | 1 in 3 | |
| | Carditis .......... | 1 | | | |
| | Pericarditis ...... | 1 | 1 | 1 in 1 | |
| | Hæmoptysis ...... | 36 | 3 | 1 in 12 | |
| | Phthisis Pulmonalis | 150 | 56 | 1 in 2$\frac{2}{3}$ | |
| | Catarrhus Acutus .. | 869 | 13 | 1 in 93 | |
| | ——— Chronicus | 345 | | | |
| | Asthma .......... | 63 | 2 | 1 in 31 | |
| | Dyspnœa.......... | 14 | | | |
| | Hydrothorax ...... | 9 | 8 | 1 in 1$\frac{1}{8}$ | |

| *Class.* | *Diseases.* | *No. treated.* | *Died.* | *Pr. of Deaths.* | *No. Deaths* |
|---|---|---|---|---|---|
| 7. Diseases of the Chylopoietic Viscera. | Gastritis | 3 | 2 | 1 in $1\frac{1}{2}$ | 22 |
| | Enteritis | 8 | 5 | 1 in $1\frac{3}{5}$ | |
| | Hepatitis Acuta | 47 | 5 | 1 in $17\frac{2}{5}$ | |
| | ——— Chronica | 40 | | | |
| | Splenitis | 16 | | | |
| | Hæmatemesis | 1 | | | |
| | Dysenteria | 42 | | | |
| | Dyspepsia | 25 | | | |
| | Colica | 47 | | | |
| | Cholera Morbus | 64 | 1 | 1 in 64 | |
| | Diarrhœa | 343 | 3 | 1 in 114 | |
| | Ascites | 17 | 6 | 1 in $2\frac{5}{6}$ | |
| | Vermes | 18 | | | |
| | Icterus | 38 | | | |
| | Obstipatio | 8 | | | |
| 8. Diseases of the Urinary Organs. | Nephritis | 6 | 1 | 1 in 6 | 3 |
| | Cystitis | 5 | 1 | 1 in 5 | |
| | Diabetes | 3 | 1 | 1 in 3 | |
| | Eneuresis | 7 | | | |
| | Gonorrhœa | 622 | | | |
| | Strictura Urethræ | 17 | | | |
| | Ischuria | 7 | | | |
| | Dysuria | 9 | | | |
| | Calculus Vesicæ | 1 | | | |
| 9. Diseases strictly local | Phlegmon | 629 | 1 | 1 in 629 | 15 |
| | Furunculus | 6 | | | |
| | Paronychya | 22 | | | |
| | Otitis | 16 | | | |
| | Epistaxis | 2 | | | |
| | Hæmorrhois | 93 | | | |
| | Hydrocele | 16 | | | |
| | Hydarthrus | 5 | | | |
| | Morbus Coxarius | 3 | 1 | 1 in 3 | |
| | Apostema Lumbare | 2 | 1 | 1 in 2 | |
| | Syphilis Primitiva | 816 | | | |
| | Ulc. Penis non Syph. | 599 | | | |
| | Bubo Simplex | 157 | | | |
| | Phymosis | 4 | | | |
| | Paraphymosis | 2 | | | |
| | Contractura | 5 | | | |
| | Hernia Humoralis | 200 | | | |
| | Sarcocele | 4 | | | |
| | Varix | 5 | | | |
| | Schirrhus | 3 | | | |
| | Tumores | 35 | 1 | 1 in 35 | |
| | Verrucæ | 21 | | | |
| | Hernia | 8 | 1 | 1 in 1 | |
| | ——— Inguinalis | 2 | | | |
| | Prolapsus Ani | 2 | | | |
| | Fistula in Ano | 6 | | | |
| | ——— in Perinæo | 1 | | | |
| | ——— Lachrymalis | 1 | | | |
| | Luxatio | 13 | | | |
| | Subluxatio | 68 | | | |
| | Vulnus | 284 | 1 | 1 in 284 | |
| | Contusio | 986 | 2 | 1 in 493 | |
| | Ambustio | 27 | | | |
| | Gelatio | 2 | | | |
| | Ulcus | 1279 | 3 | 1 in 426 | |
| | Necrosis | 8 | | | |
| | Fractura | 44 | 4 | 1 in 10 | |
| | Amputatio | 3 | | | |
| | Punitus | 483 | | | |

TABLE, No. III.

*Shows the mean Annual Strength of the Troops; the average Proportion of Sick per Cent.; the Number of Deaths; and the Proportion of Deaths per Cent. of the mean Strength during Seven Years divided into annual Periods.*

| *Years.* | *Mean Strength.* | *Average Proportion of Sick per Cent.* | *Number of Deaths.* | *Proportion of Deaths per Cent. of the mean Strength.* |
|---|---|---|---|---|
| 1816 | 2591 | 4.7 | 38 | 1. 4 |
| 1817 | 3143 | 3.2 | 19 | 0. 6 |
| 1818 | 2939 | 3.5 | 19 | 0.61 |
| 1819 | 2714 | 3 | 33 | 1. 2 |
| 1820 | 4853 | 3.3 | 61 | 1. 2 |
| 1821 | 2903 | 3.5 | 39 | 1. 3 |
| 1822 | 1680 | 4.4 | 21 | 1. 2 |

TABLE, No. IV.

*Shows the mean Result of Seven Years.*

| *Strength.* | *Proportion of Sick per Cent.* | *Annual Deaths* | *Proportion of Deaths per Cent. of the mean Strength.* |
|---|---|---|---|
| 2975 | 3.6 | 33 | 1.1 |

By this table it appears that, on an average of seven years, there has been one death in ninety of the whole strength.

The preceding tables possess much interest, on account of the certainty of the data from which they are framed. They may serve for making comparative statements with respect to the prevalency of particular diseases, the proportion of sick, and the ratio of mortality in this country. Dr. Hennen and Dr. Erly have, in their half-yearly reports, so comprehensively and so ably detailed the history of the health of the troops in Scotland, during the greater portion of the septennial period included in the tables, that it would be presumption in me to return to that subject, except in a very general way. The few remarks I intend to make will chiefly relate to the relative mortality of diseases of the thorax, when compared to the fatality occasioned by diseases of other regions of the body. Pectoral complaints are the only class of diseases to which much attention has been paid, with regard to their comparative frequency or rarity of occurrence, during particular portions of time and in different countries.

I may here observe, that some of the specific names of diseases, inserted in the returns for the years 1816 and 1817, are rather indefinite,—such as Hydrops, Morbi Venerei, and Morbi Chronici. The reports for the succeeding years are, however, unexceptionable, being compiled according to the "Table of Diseases for Medical Returns."

By the returns it appears that a very unusual number of cases of intermittent fever were admitted into the hospital during the year 1816. This circumstance is to be ascribed to the arrival of the 78th Regiment in this country from Belgium, where it had been employed, and where many of the men had suffered severely from endemic fever; a disease to which they continued very liable for some time.

For several years included in the septennial period, contagious fever prevailed in Scotland; and, although the troops were supposed to suffer less by it than the inhabitants, yet a considerable number fell under its influence, as appears by the returns.

An inspection of Table No. II. will show that nearly one-half of the deaths which took place occurred from diseases of the chest. Perhaps it may be inferred that a much greater number of these cases suffered from tubercular disease of the lungs, than are arranged under the head Phthisis Pulmonalis. In all likelihood, not a few of the fatal cases under the heads Pneumonia, Hæmoptysis, Catarrhus, and Hydrothorax, would have been more appropriately classed under that disease.

To give the returns of the sick of the army all the accuracy and precision of which they are capable, the denomination of the diseases of patients ought to be changed according as the symptoms would warrant. Patients who are admitted with symptoms of pneumonia or catarrh, and their disease classed under one of these heads, sometimes subsequently evince appearances of consumption. Were cases of this kind discharged, and re-admitted under phthisis pulmonalis, the designation of the fatal disease would be more accurate. It may become a question whether the opinions entertained of the diagnosis of a disease during life might not be corrected by post-mortem examinations. The returns may be consulted, and inferences drawn from them with regard to the fatal diseases, or causes of death, without paying due attention to examine the history of the case, and the appearances on dissection. It is therefore desirable that the numerical statements should be as exact as they can be made, and to require as little collateral explanation as possible. I am aware that the examination of bodies after death occasionally discover traces of disease of so complicated a nature, that some doubt may be entertained regarding the immediate cause of death; or, in other words, under what head the disease should be classed. Cases of so ambiguous a nature are not, however, frequent.

In this country, the fatal results among the troops are chiefly from diseases of the thorax, whereas, in warm climates, they principally occur from constitutional affections, as fever, and from diseases of the chylopoietic viscera. To compare the Sick-returns of the army employed in different countries, affords much useful information regarding the influence of climate upon the human constitution.

It becomes an interesting question for us to ask, whether the troops employed in Great Britain are as liable to phthisis pulmonalis as the non-military inhabitants? Sir James M'Gregor, in his "Sketch of the Medical History of the British Armies in Spain and Portugal," informs us that "in England this disease (phthisis pulmonalis) is as frequent in the army as it is in civil life." In the same valuable paper he states, that, "under ordinary circumstances, and when no epidemic or contagious disease prevailed, I found, in England, the deaths from consumption to amount to one-fifth, one-fourth, and in some regiments even as high as one-half of the mortality.

Presuming that almost all the fatal cases stated to have followed diseases of the thorax died in consequence of tubercular disease of the lungs, the proportion of cases of consumption will be very nearly one-half of the mortality; or, to be more specific, as 107 is to 123.

What proportion does this ratio of deaths by consumption bear to that which occurs in civil life among males between the ages of twenty and forty? I am not aware of any well-constructed tables that show the proportion of deaths in civil life by consumption, during particular ages. Data of this kind are necessary before an accurate comparison can be instituted between the mortality which occurs in the army and that of the inhabitants. Numerous interesting documents have, however, been published in regard to the proportional fatality by consumption during all ages; and, although many of these statements are compiled from uncertain data, by which means such correct inferences cannot be drawn from them as may be obtained from military returns, yet are they well worthy of our attention.

At Chester, the deaths by consumption in two years were 135 out of 731, or 10 out of 54. By the London Bills of Mortality, from 1796 to 1799, it appears that 52,237 died, exclusively of casualties and children still-born. Of the above number, 17,559 died of consumption, being somewhat more than one-third of the whole deaths. At the end of the eighteenth century, Dr. Heberden states the proportion of deaths by consumption, compared with the number of deaths by other diseases, as 1 to 4.2.

The annual average of deaths for seven years, in Bristol, was

236, and the average deaths by consumption, 98; being about one death by this complaint for every $2\frac{1}{2}$ by other diseases.

In America, the proportion of deaths by consumption appears to be almost as high as in Great Britain. Nearly one-fourth of the deaths in New York is said to be occasioned by consumption, or in the proportion of 395 to 1717; which is as one to 4.3. By the Philadelphia Bills of Mortality for the year 1815, it appears that the total number of deaths was 2040, of which 347 died of consumption; being nearly in the ratio of one in six in all ages: between the ages of ten and forty, the proportion was about one in three. Spalding reports the annual mortality at Portsmouth, New Hampshire, by consumption, to be one in five of the whole deaths.

Consumption appears to be more frequent in Great Britain than on the continent of Europe. The proportional estimate has been rated at one-fourth of the whole deaths in this country, one-fifth in Paris, and one-sixth in Vienna. Bayle, whose authority cannot be doubted, asserts that, of 500 who die in the hospitals at Paris, 100 die of phthisis; and, of the other 400, at least 50 are complicated with phthisis. But, perhaps, the simplest and most satisfactory mode of estimating the fatality of a particular disease, is to state the proportion of deaths by the complaint to the whole number of inhabitants, not to the number of deaths. Thus, in Great Britain, it is estimated that one in 220, of all ages, dies annually by consumption.*

In Geneva, Dr. Chisholm informs us that one dies annually by this disease in 521. The result of six years' observation among European soldiers, in the interior provinces of the island of Ceylon, shows that one died annually of consumption in 1220. How very different from this is Dr. Sinclair's statement in regard to the prevalence of pectoral complaints among the seamen belonging to the royal navy employed in the Mediterranean. From his reports, as stated by Dr. Clarke, it appears that, during the years 1810, 1811, 1812, there were admitted into the Naval Hospital in the Mediterranean, from the fleet, (which consisted of 30,000 men,) 455 cases of phthisis pulmonalis, and 140 of pneumonia; being an annual proportion of one in 195 affected with the former complaint, or of one in 150 when the two diseases are taken together. From the fatal tendency of phthisis pulmonalis, we may infer that those individuals who were attacked soon became its victims.

The average age of seamen and soldiers being nearly alike, statements regarding the prevalence of disease among them admit of a closer degree of comparison than among more dissimilar classes of society. By the above tables it will appear that,

---

* Dr. Woollecombe.

among the troops employed in Scotland, the annual proportion of deaths by diseases of the thorax, is to the whole strength as one in 198.

Much obscurity still exists with respect to the remote causes of consumption. There are, however, some striking and well-ascertained facts with respect to its natural history, (if I may be permitted to use the expression,) which merit our regard. Europeans are much less liable to phthisis in tropical countries than in high latitudes. But it is difficult to know what we should infer from this fact, because negroes are very obnoxious to consumption in intertropical climates; and Malays are less exempted from it than natives of Europe. In India, the Half-caste race are extremely liable to it.

Among Europeans, it appears to be chiefly prevalent between the 45th and 60th parallels of north latitude. We are informed that it is little known in Russia and towards the arctic regions. But, although the habitat of a disease may be known, the actual cause may be still involved in the most complete obscurity.

Great transitions of temperature, particularly from heat to cold, are often assigned as the exciting cause of consumption; and perhaps numerous cases occur where the disease is, to a certain degree, attributable to mutations of weather.

Many circumstances must, however, be taken into account before a due estimate can be made with regard to the influence of a reduction of temperature in exciting pulmonic diseases. Our sense of heat and cold being merely relative, the change of temperature indicated by a thermometer is but an imperfect test of variation, as it affects the human system. In warm climates, a reduction of temperature to the extent of eight or ten degrees, by means of rain and wind, occasions unpleasant sensations of cold, even although the thermometer may indicate a temperature not below 74° or 76°. Perhaps a decrement of ten degrees of temperature in the torrid zone, is as much felt by the human system as a reduction of twenty degrees is in the latitude of 50° and upwards.

Sudden variations of temperature appear to be more apt to cause pulmonic diseases, than when the change, although great, is gradual. The thermometrical vicissitudes of temperature of the atmosphere are often considerable in equatorial regions, yet consumption is comparatively rare in climates where the mean heat exceeds 70° Fahrenheit. The difference of temperature between the atmosphere during night in warm climates, and that of the earth exposed to the sun at mid-day, is sometimes as great as eighty degrees, which is a greater variation than commonly occurs in temperate climates. The variation of temperature, although great, in tropical climates, is seldom sudden, unless under particular circumstances of exposure, &c.

In all probability, many of the bad consequences occasioned by a variation of temperature arise from the sudden transition from apartments, heated perhaps as high as 65° or 70°, to an atmosphere of about 35° or 40°, much aided in its cooling effects upon the system by aerial currents. Unless where the variation is rapid, the human body will bear considerable vicissitudes of temperature without much danger.

It is very remarkable that the application of the same causes of disease,—namely, great and sudden reduction of temperature, by being exposed to rain, and sitting in wet clothes, &c.—produce different effects in different climates. In this country, pulmonic disease is a frequent result; but, in tropical regions, the consequences to be feared from such an exposure would be remittent fever, abscess in the liver, or inflammation and consequent gangrene of the villous coat of the large intestines.

It has been stated by some authors, that individuals who follow particular trades are remarkably liable to consumption: hence they infer that the occurrence of the disease is often more or less connected with certain occupations. Dryers of flax and feathers, pin-makers, stone-cutters, &c. are said to be peculiarly obnoxious to pulmonic complaints. The same is stated of weavers, and others who follow similar sedentary trades. It has likewise been asserted that the indigent are more liable to consumption than the superior classes of society, who are better fed, more comfortably clothed, and less exposed to transitions of temperature. How far these conjectures are founded in truth, we are at a loss to know, for want of comparative statements, or accurate resources from which they might be framed.

The influence of some of the circumstances to which the prevalence of consumption have been ascribed among the civil inhabitants, might be better ascertained by comparing its fatality among certain classes of individuals, or trades, with its frequency in the army, which is composed of a class of men carefully selected, and deemed free from any striking tendencies to pulmonic complaints, well clothed and occupying good quarters, sufficiently nourished; not more intemperate than individuals of their own rank in society; rarely exposed, during peace, to sudden transitions of weather; little affected by mental disquietude, or under the influence of the depressing passions.

Notwithstanding the care with which this subject has been investigated by medical observers of all ages, the nature of the influential cause of that kind of inflammation of the lungs which is accompanied with, or followed by, the formation of tubercles, is still but very imperfectly known. The same may be said of the circumstances which occasion gangrene of the villous coat of the large intestines, giving rise to dysenteric symptoms in tropical climates,—perhaps more particularly in India.

It is not evident that consumption is so intimately connected with a very low temperature, or even with considerable transitions of weather, as would at first sight appear. According to Bayle, "in every season nearly an equal number die of phthisis." More light might, perhaps, be thrown upon the remote cause of this disease, were accurate and comprehensive tables kept of the nature of the fatal diseases which occur among the inhabitants of a country, proportion of mortality, &c. By this means we might ascertain the class of inhabitants among which consumption prevailed most; how far particular trades occasioned the complaint; whether, and to what degree, the poor are more liable to it than people in easy circumstances. From well-established facts of this kind, some information regarding the best means of preventing phthisis, or, what is nearly the same thing, avoiding its exciting causes, might perhaps be deduced. More benefit is likely to be derived from endeavours to prevent the disease, than by establishing new plans of treatment. But, before much can be done on this important subject, proper data are wanted to reason upon. "Solitary facts are seldom of much real importance: it is by having the power of multiplying, contrasting, comparing, and combining these, that material progress can be made in any science." (Dr. Barron's *Translation of* Bayle *on Consumption.)*

Tables Nos. III. and IV. show the average proportion of sick, and the average proportion of deaths per cent. of the whole strength. The mean proportion of sick for seven years amounts to 4.4, a ratio extremely small, when we consider that it includes every man, however trivial his complaints may be, who is not completely fit for duty.

In tropical climates, the proportion of sick is seldom below six, and frequently it rises as high as twelve or fifteen per cent. From a mean of seven years, it appears that the mortality among the troops in Scotland amounted to 1.1 per cent, or one in ninety of the whole strength. This is a less ratio of mortality than Sir Gilbert Blane has estimated the mortality of subjects from twenty to forty to be in Great Britain,—namely, one in eighty. The correctness of his estimate is, however, strongly corroborated by the preceding tables.

May we not infer that the mortality of the troops employed in the East and West Indies, is eight times greater than what occurs among troops on duty in Great Britain. We are not, I think, warranted in estimating the mean fatality of troops employed in equatorial climates, at a less annual ratio than from eight to nine per cent.

With the view of contrasting the ratio of mortality among troops in this country with that which happens in tropical climates, I shall subjoin a return of the 78th Regiment, for a period of nineteen years, while this corps was employed in India, or in the island of Java; and an account of the mortality that happened among the troops on duty in the West Indies, for a period extending to nineteen years, or from 1796 to 1814 inclusive.

RETURN *of the mean Strength of the First Battalion of the* 78*th Regiment, (exclusively of commissioned Officers;) the Number of Deaths; the Proportion of Deaths by Disease per Cent. per Annum; and an Account of the Stations where the Corps was employed, from its Arrival in India,* 16*th February,* 1797, *till the* 24*th December,* 1815.

| *Years.* | *Mean Strength.* | *Killed in the Field.* | *Died by Disease.* | *Proportion of Deaths per Cent.* | *Where employed.* |
|---|---|---|---|---|---|
| 1797 | 1149 | .. | 115 | 10 | Fort William and Birhampore. |
| 8 | 1010 | .. | 79 | 7.8 | Allahabau and Caunpore. |
| 9 | 974 | .. | 58 | 8.9 | Caunpore and Fort William. |
| 1800 | 952 | .. | 53 | 5.5 | Fort William. |
| 1 | 951 | .. | 45 | 4.6 | Fort William. |
| 2 | 940 | .. | 78 | 8.3 | Fort William. |
| 3 | 857 | 47 | 119 | 13.8 | In the Field. |
| 4 | 744 | .. | 145 | 19.4 | Bombay. |
| 5 | 694 | .. | 80 | 11.5 | Bombay. |
| 6 | 749 | .. | 38 | 5 | Bombay. |
| 7 | 677 | .. | 24 | 3.2 | Island of Java. |
| 8 | 781 | .. | 34 | 4.3 | ...... |
| 9 | 825 | .. | 54 | 6.5 | ...... |
| 10 | 1064 | .. | 43 | 4 | ...... |
| 11 | 941 | 28 | 184 | 19.5 | At sea, and in the Island of Java. |
| 12 | 748 | 6 | 192 | 25.6 | Java. |
| 13 | 690 | .. | 81 | 11.7 | Java. |
| 14 | 684 | .. | 80 | 11.6 | Java. |
| 15 | 644 | .. | 33 | 5.1 | Java. |

Mean annual proportion of deaths per cent. 9.6. Presuming that the vacancies were filled up as they occurred, according to this ratio of mortality a regiment would require to be replaced every 10.4 years.

The elements of the above returns were extracted from Sir T. S. Raffles' "History of Java."

TABLE *of* DEATHS *by* DISEASE *in the* BRITISH ARMY *serving in the West Indies.*

| EUROPEANS. | | | |
|---|---|---|---|
| *Years.* | *Medium Monthly Returns.* | *Died.* | *Deaths per Cent. of the whole Strength.* |
| 1796 | 19.676 | 6.484 | $40\frac{1}{4}$ |
| 7 | 13.626 | 3.766 | $32\frac{3}{4}$ |
| 8 | 9.120 | 1.602 | $17\frac{1}{2}$ |
| 9 | 7.684 | 876 | $11\frac{1}{4}$ |
| 1800 | 8.340 | 1.221 | $15\frac{1}{2}$ |
| 1 | 11.745 | 2.340 | $22\frac{1}{4}$ |
| 2 | 10.198 | 990 | 11 |

Mean annual deaths per cent, 21.3. This ratio of mortality approaches nearly that which occurred during an average of three years in the 73d Regiment, while it was employed in Ceylon,—namely, 25.5 per cent. According to the proportion of mortality stated in the above table, a regiment, or body of troops, would require to be replaced every 4.7 years.

The above table is copied from Bolingbroke's "Description of the Settlement of Demarara." Upon what authority it is compiled, I am ignorant.

The following Returns of the mortality of the army serving in the Windward and Leeward Islands, and British colonies on the coast of America, is merely an alteration of the form of Dr. Robert Jackson's "Comparative Return," as inserted in the second part of the "Transactions of the Medical Society of London." Not having had access to the volume of the Transactions in which Dr. Jackson's paper is published, I have copied the result from a periodical work.

| *Years.* | | *Proportion of Mortality per Cent. of the Strength.* |
|---|---|---|
| 1803 | .... | 10.5 |
| 4 | .... | 18.1 |
| 5 | .... | 19.3 |
| 6 | .... | 11.1 |
| 7 | .... | 9.7 |
| 8 | .... | 15.8 |
| 9 | .... | 13.8 |
| 10 | .... | 20.5 |
| 11 | .... | 12.5 |
| 12 | .... | 8.2 |
| 13 | .... | 6.8 |
| 14 | .... | 5 |

Mean annual proportion of deaths per cent. 12.6. According to this ratio of mortality, the troops serving in the West Indies will require to be replaced every 7.9 years.

To the above abstract of the Sick-returns of the troops employed in North Britain, I have added a statement of the number of recruits for the army, inspected at Edinburgh, since the 25th March, 1817.

RETURN *of the* NUMBER *of* RECRUITS *for the* ARMY, *inspected at Edinburgh, from the 25th March,* 1817, *to the* 31*st December,* 1822, *divided into annual Periods; wherein the Number deemed fit for the Service are distinguished from those considered to be unfit, with a Specification of the Causes of Rejection.*

| | *From 25th March* 1817 *to 31st Dec.* | 1818 | 1819 | 1820 | 1821 | 1822 |
|---|---|---|---|---|---|---|
| Deemed fit for the Service .... | 521 | 466 | 401 | 729 | 402 | 765 |
| Deemed unfit for the Service .. | 140 | 148 | 196 | 198 | 227 | 276 |
| Number inspected ...... | 661 | 614 | 570 | 927 | 629 | 1041 |
| *Causes of Rejection.* | | | | | | |
| Marks of Punishment .......... | .. | 17 | 15 | 7 | 15 | 8 |
| General Debility, or unsoundness of Constitution .............. | 13 | 18 | 19 | 16 | 43 | 38 |
| Cutaneous Diseases ............ | 3 | 2 | 3 | 3 | 9 | 16 |
| Ulcers, or Cicatrices of Ulcers .. | 19 | 16 | 17 | 21 | 38 | 23 |
| Scrofula .................... | 5 | 9 | 9 | 5 | 5 | 5 |
| Various Diseases of the Eyes impairing Vision ............... | 9 | 3 | 6 | 7 | 7 | 16 |
| Strabismus .................. | .. | .. | .. | .. | 1 | 5 |
| Deafness .................... | .. | .. | .. | .. | 1 | .. |
| Dis$^{ed}$. Teeth, or loss of many teeth | 3 | .. | .. | .. | 5 | 9 |
| Artificial Teeth .............. | .. | .. | .. | .. | 1 | .. |
| Polypus of the Nostril ........ | .. | .. | .. | .. | .. | 1 |
| Malformation of Chest ........ | 2 | 5 | 4 | 3 | 20 | 18 |
| Malformation of Spine ........ | 5 | 2 | 9 | 26 | 9 | 6 |
| Injuries of the Hand or Fingers, including Contractions and Mutilations of ................ | 5 | 5 | 12 | 13 | 8 | 8 |
| Inguinal Rupture, or disposition to Rupture ................ | 6 | 4 | 3 | 1 | 9 | 6 |
| Diseased Hip-joint ............ | 1 | .. | .. | .. | .. | .. |
| Gonorrhœa .................. | .. | .. | .. | .. | 1 | .. |
| Varicose State of the Right Testicle, or Spermatic Cord .... | 2 | 3 | .. | .. | .. | 1 |
| The same of the Left Testicle, or Spermatic Cord ............ | 13 | 23 | 12 | 43 | 10 | 31 |
| —— of both Testicles, or Cords | .. | 1 | .. | 1 | .. | .. |
| Right Testicle not excluded .... | .. | 1 | .. | .. | .. | .. |
| Left Testicle not excluded .... | .. | .. | 1 | 1 | .. | 2 |
| Diseased Testicles ............ | 7 | 2 | 7 | 2 | 2 | .. |
| Hydrocele .................... | 1 | .. | .. | .. | 1 | 2 |
| Diseased Knee-joint .......... | 1 | 1 | .. | .. | 1 | .. |
| Much In-knee'd ................ | 2 | .. | .. | .. | .. | .. |
| Varicose Veins of the Right Leg .......... | 4 | .. | 3 | 6 | 9 | 13 |
| Varicose Veins of the Left Leg .......... | 7 | 12 | 17 | 14 | 11 | 11 |
| Varicose Veins of the Left Leg and Left Testicle, or Cord ...... | .. | 5 | 4 | 4 | 2 | 2 |
| Varicose Veins of both Legs ............ | 14 | 8 | 12 | 13 | 8 | 9 |
| Varicose Veins of both Legs and Testicles, or Spermatic Cords .... | .. | 1 | .. | .. | .. | .. |
| Varicose Veins Of Right Leg and Left Testicle, or Cord .......... | .. | 1 | 2 | .. | .. | 1 |
| Diseased Legs or Feet, including Malformations and Mutilations | 18 | 9 | 13 | 5 | 6 | 10 |
| Flatness of the Soles of the Feet | .. | .. | 1 | 7 | 5 | 33 |
| | 140 | 148 | 169 | 198 | 227 | 274 |

